# A composite CdS thin film/TiO_2_ nanotube structure by ultrafast successive electrochemical deposition toward photovoltaic application

**DOI:** 10.1186/1556-276X-9-631

**Published:** 2014-11-25

**Authors:** Han Fu, Hong Liu, Wenzhong Shen

**Affiliations:** 1 Department of Physics, Shanghai Jiao Tong University, 800 Dong Chuan Road, Shanghai 200240, People’s Republic of China

**Keywords:** Composite tubular structure, CdS thin film, TiO_2_ nanotube, Ultrafast, Successive electrochemical deposition, Solar cell

## Abstract

Fabricating functional compounds on substrates with complicated morphology has been an important topic in material science and technology, which remains a challenging issue to simultaneously achieve a high growth rate for a complex nanostructure with simple controlling factors. Here, we present a novel simple and successive method based on chemical reactions in an open reaction system manipulated by an electric field. A uniform CdS/TiO_2_ composite tubular structure has been fabricated in highly ordered TiO_2_ nanotube arrays in a very short time period (~90 s) under room temperature (RT). The content of CdS in the resultant and its crystalline structure was tuned by the form and magnitude of external voltage. The as-formed structure has shown a quite broad and bulk-like light absorption spectrum with the absorption of photon energy even below that of the bulk CdS. The as-fabricated-sensitized solar cell based on this composite structure has achieved an efficiency of 1.43% without any chemical doping or co-sensitizing, 210% higher than quantum dot-sensitized solar cell (QDSSC) under a similar condition. Hopefully, this method can also easily grow nanostructures based on a wide range of compound materials for energy science and electronic technologies, especially for fast-deploying devices.

## Background

Nanostructure-based semiconductors have attracted continuous attention and inspired numerous novel results on material and structure studies. On the one hand, compound semiconductors such as CdS [1-3], CdSe [4-6], CdTe [7,8], and PbS [9] have been extensively studied as photon absorbers due to their excellent performance in solar energy conversion, photodetectors, and photocatalysis [10-13]. On the other hand, nanostructure like nanotube arrays, nanoporous films, and nanorods (mostly by semiconducting oxides, TiO_2_, ZnO, etc.) have also become promising materials for their unique optoelectronic characteristics [14,15] and advantage to load various objects (molecules, quantum dots, etc.) [16,17]. Among them, TiO_2_ nanotube arrays (NTAs) have shown their potential advantages on better electron transport ability and feasibility for morphology control. Combining them, quantum dot-sensitized solar cells (QDSSCs) have been developed and continuously improved due to their low cost, low material requirement, and convenience for fabrication in photovoltaic application and scientific research [18,19]. Nevertheless, it still remains a challenging question to develop novel functional nanostructures to significantly improve the photovoltaic devices or other optoelectronic devices without complicated procedures or expensive and toxic materials. Taking example on the CdS QDSSCs on TiO_2_ NTA structure, one possibility is to replace the quantum dots (QDs) with a coaxial tubular structure of CdS, which can hopefully increase the amount of photo absorber and diminish the size effect of quantum dots [20] and, thus, significantly increase its conversion efficiency.

For this purpose, the primary task is to seek for the suitable growth method. Currently, the main methods to fabricate complex structures include atomic layer deposition (ALD) [21-23], chemical bath deposition (CBD) [24,25], successive ionic layer adsorption and reaction (SILAR) [26-28], and electrochemical deposition (ECD) [29-31]. In spite of their own advantages, they have also met limitations in small sample size (ALD), slow growth rate (ALD, CBD, ECD), and complicated procedures (SILAR), which have limited their adaptability in real applications. Moreover, when trying to grow compound semiconductors in situ on nanostructured substrate, more challenges would emerge [22,32-35], including the control of material quality (chemical composition, morphology, and microstructure) and the combination of deposited material and targeted substrate. For instance, in CdS deposition on nanotube structures like TiO_2_ NTAs [2], the inhomogeneity and discontinuity of the material amount along the depth of the tubes [36] have often appeared, while the sensitivity of the deposition condition may also lead to unwilled island formation and defects [37], overabundance of components, and possible etching effect on the TiO_2_ NTA substrate [38,39].

To increase the deposition rate, some comprehensive ways might be normally considered, including the increase of precursor concentration [40], applying fast reaction systems [32], and increasing the reaction temperature [41]. However, those means would meet significant difficulties when trying to effectively a grow material inside nanostructures, e.g., nanotubes [4]. As mentioned before, the influence of viscosity, concentration gradient of reaction species, and blocking effect by early grown particles in the solution or on tube openings would significantly deteriorate the amount, homogeneity, and quality of the material grown inside the nanostructures. Moreover, to enhance the combination of the deposited material, the choice of suitable deposition medium (gas or liquid phase) and the addition of surfactant were also carefully considered, though they would further increase the complexity of the fabrication [42]. Therefore, a method would be needed for functional nanostructure fabrication which combines high efficiency, simplicity, and adequate freedoms of growth manipulations. Taking example on CdS growth in TiO_2_ NTAs, a possible highly efficient but simple system would be in solution via a chemical reaction among simple precursors. It is also better to use a simple solution as possible and exclude or reduce the use of surfactant. Furthermore, a modulated external electric field would be useful to manipulate the reaction without the involvement of new complicated factors. Finally, to annihilate the influence of early reactions, the simultaneous introduction of precursors can possibly be replaced by subsequent introduction. In previous works, we have studied the reaction-diffusion system in the TiO_2_ NTAs formation and, later, the in situ growth of PbS quantum dot in them, which has offered preliminary basis for this purpose [43].

In this work, we represent a uniform and ordered CdS thin film (CTF)/TiO_2_ nanotube (TNT) structure by an in situ successive electric-field-assisted chemical deposition method in an aqueous solution under room temperature (RT). CdS film with 18 to 28 nm thickness was grown in NTAs in about 90 s, more than two orders of magnitude faster than the normal method. Only simple precursors (Cd(NO_3_)_2_ and Na_2_S) were applied and successively supported (Cd(NO_3_)_2_) from injectors. The as-grown film has good homogeneity and continuity inside the NTAs as well as chemical purity (almost 1:1 component ratio of Cd and S in the as-formed layer). This was aided by the application of AC voltage instead of constant voltage. Furthermore, we have discovered that the microstructure of the CdS film can be changed by adjusting the applied voltage magnitude. The as-formed CTF/TNT complex structure was then installed into a sensitized solar cell to characterize its optoelectronic properties. It has shown relatively strong conversion in a wide range of 500 to 600 nm (corresponds to photon energy 2.4 to 2.0 eV), which could be an advantage for matching the solar spectrum. The CTF/TNT-based back-side-illuminated sensitized solar cells (without post chemical treatment) have shown conversion efficiency up to 1.43%, 210% higher than that of the QDSSC based on the same system, together with a short circuit current increase of 135%. Continuous growth system could be further improved if feedback flow control devices and micropumps are applied in the injection site. Hopefully, this method would introduce a new low cost and controllable way to fabricate not only CdS but also many new nanostructured materials and devices based on them with rapid deployment.

## Methods

### Fabrication of TiO_2_ nanoporous (NP) films and NTAs

For TiO_2_ NP films, 2.4 g P25 powder (Degussa P25, Evonik Industries, Essen, Germany) was mixed with 0.75 g ethylcellulose (46 cp, Sigma-Aldrich, St. Louis, MO, USA) in 9 ml terpineol (99.5% purity, Sigma-Aldrich), and then coated onto the fluorine-doped tin oxide (FTO) substrate. Afterwards, it was annealed for 1 h at 500°C in air. For TiO_2_ NTAs, a two-step anodization process was applied on thin Ti sheets (0.25 mm thick, 99.7% purity, Sigma-Aldrich). The Ti sheets were cleaned with ethanol, acetone, and isopropanol in sequence for 30 min each. All the processes were carried out under an ultrasonic bath to remove possible contamination. The first anodization was carried out at constant voltage (60.0 V) in the solution of 0.35 wt% of NH_4_F and 3.00 wt% of H_2_O in ethylene glycol (E.G.) at 5°C for 4 h. The second anodization was performed at 60.0 ± 1.7 V at 5°C for 30 min in the same solution. The as-formed TiO_2_ NTAs were annealed in air at 500°C after being rinsed with deionized water and ethanol. It will serve as principal substrates for the following experiments, while the NP films will be used for preliminary research.

### In situ growth of CdS on substrates

For the preliminary experiments of CdS deposition on NP films and NTAs, the substrates were immersed vertically into the electrolyzing cell containing 0.3 M of CdCl_2_ and 0.06 M of Na_2_S_2_O_3_. A standard Pt electrode was applied as the counter electrode. The substrate was negatively biased by constant DC voltage ranged from 1.5 to 2.5 V. Experiments were carried out in 200-ml deionized water at 50°C for 2 h. The pH of the solution was maintained at 3.0 during the reaction by the titration of vitriol and monitored by a pH meter (SevenMulti S40, Mettler-Toledo International Inc., Schwerzenbach, Switzerland).

For the successive deposition of CdS thin film in NTAs with manipulated voltage, NTAs were immersed vertically into the electrolyzing cell containing 200 ml aqueous solution of Na_2_S (0.001, 0.003, 0.005 M). Three types of bias voltage were applied on the substrate: constant, impulse (*T* = 4 s, can be found in the Additional file 1), and AC (*T* = 4 s, square wave, with an equal height of the cathodic and anodic pulses, can be found in the Additional file 1), with an absolute magnitude from 2.0 to 10.0 V. During the experiment, 100 ml aqueous solution of Cd(NO_3_)_2_ (0.001, 0.003, 0.005 M, kept the same with Na_2_S) was slowly injected into the electrolyzing cell by a syringe at the rate of 1.0 ml/s. The reaction temperature was at RT and time ranged from 1 to 1,800 s.

### Composing of solar cells

The configuration of the PV test in this work was based on back-side-illuminated sensitized solar cells. The as-fabricated CdS-coated TiO_2_ NTAs (with area of 0.25 cm^2^) was mounted together with a CuS counter electrode, which is suitable for polysulfide electrolyte in back-side illumination mode [44]. To prepare the counter electrode, the FTO glass was firstly coated with Cu from a Cu source (99.9%) by magnetron sputtering (with a pressure of 1 Pa and a power of 60 W) at RT for 10 min and then loaded into a 100-ml Teflon-lined autoclave with 50 ml ethanol and 0.03 g excessive sulfur powder. The autoclave was kept at 60°C for 12 h and then the as-formed CuS electrode was cleaned by ethanol and dried in air. The polysulfide electrolyte contained 1 M Na_2_S and 1 M sulfur in a mixed solution of methanol and water with the volume ratio of 7:3.

### Characterization of as-fabricated materials and solar cells

The morphology and distribution of CdS thin film into TiO_2_ NTAs were characterized by field emission transmission electron microscopy (TEM, JEM-2100F, JEOL Ltd., Tokyo, Japan). The surface morphology of samples was characterized by field emission scanning electron microscopy (FE-SEM, JEOL JSM). The element analysis was carried out by energy dispersive X-ray (EDX) during the FE-SEM observation. Reflection spectra and quantum efficiency were recorded by Solar Cell Quantum Efficiency Measurement System (QEX10, PV Measurements, Inc., Boulder, CO, USA). The photocurrent density - photovoltage (J-V) characteristics of the as-fabricated solar cell was measured under AM1.5 (100 mW cm^-2^) illumination provided by a solar simulator (Oriel Sol-2A, Newport, Irvine, CA, USA) with a Keithley 2400 source meter (Keithley Instruments Inc., Cleveland, OH, USA).

## Results and discussion

**Figure 1:**
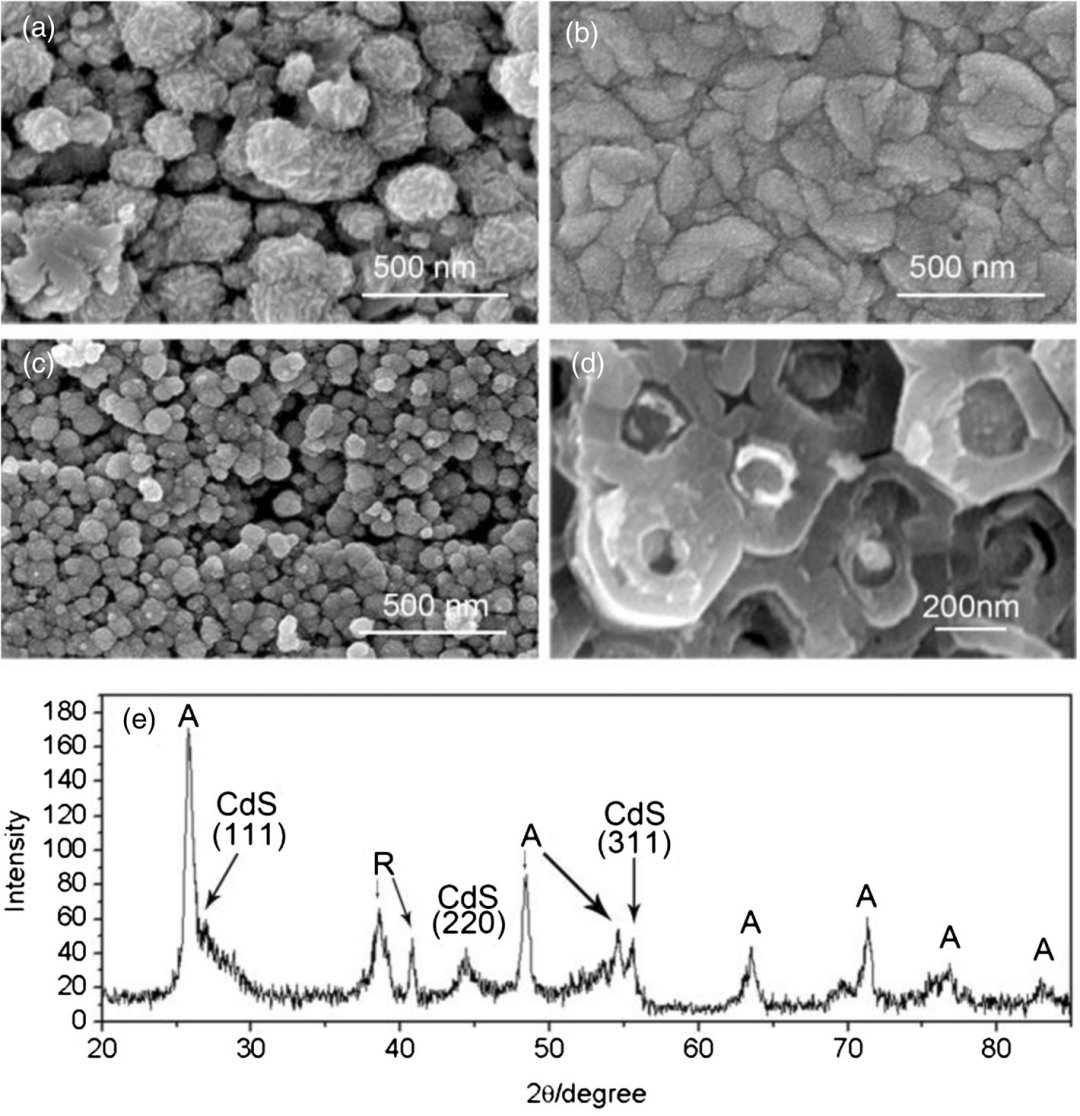
**CdS deposition by EACBD on TiO**_**2 **_**NP film and on NTA substrates. (a)** CdS deposition on TiO_2_ NP at -1.9 V. **(b)** CdS deposition on TiO_2_ NP film at -2.1 V. **(c) (d)** CdS deposition on TiO_2_ NTAs at -2.1 V: **(c)** top view and **(d)** sectional view, from the same angle. **(e)** XRD pattern of the CdS-coated TiO_2_ NTAs.

First of all, conventional electrochemical deposition method was applied to preliminarily investigate the CdS growth on TiO_2_ NP films and in NTAs. The precursors (CdCl_2_ and Na_2_S_2_O_3_) were introduced into the reaction system under potentiostatic condition and other parameters (precursor type, solution, and temperature) similar to the conventional method at 50°C [45,46]. Due to the reaction mechanism, the substrate was negatively biased by a voltage from -1.5 to 2.5 V. As a result, the significant deposition of material took place in the experimental range (the detailed conditions of deposition can be found in Table 1 at the end of this article). As shown in Figure 1a,b, as-deposited material has the form of loosely packed round particles (size of about 50 to 250 nm) at -1.9 V and more continuous film composed of anisotropic grains at -2.1 V, respectively. It indicates that the morphology of the as-formed deposited material is very sensitive to the voltage magnitude. Based on that, similar processes were conducted on TiO_2_ NTA substrate at -2.1 V, as shown in Figure 1c,d. The particles of a smaller size have been formed on top as well as inside the tubes. Figure 1e displays the XRD patterns of the CdS/TiO_2_ NTAs. Subsequent XRD measurement in Figure 1e has shown characteristic peaks at 26.4°, 43.9°, and 52.0°, corresponding to the (111), (220), and (311) crystallographic planes of CdS nanocrystals, respectively. According to EDX measurement, the contents of Cd and S on the NP substrate were Cd 1.83% and S 0.45%, while the ones on the NTAs were Cd 13.19% and S 1.98%, respectively. It implied that only a part of the as-formed material in the NTAs was CdS (detailed values can be found in Additional file 1: Table S1). This is understandable considering the repulsing and attracting effect of the electric field on the main reacting anions and cations (Cd^2+^ and S_2_O_3_^2-^), respectively [31].

**Table 1:** Conditions of deposition in different experiments

**Experiment**	**Precursors (M)**		**Overvoltage (V)**	**Current density (A cm**^**-2**^**)**	**Period (s)**	**Group**
			-1.9	-5.66182*10^-2^	Inf.^a^	1
Method and substrates	Na_2_S_2_O_3_(0.3)	Na_2_S_2_O_3_(0.06)	-2.1	-7.56863*10^-2^	Inf.^a^	2
			-2.1	-9.06196*10^-2^	Inf.^a^	3
Forms of voltages (injection)	CdS (0.001)	Na_2_S (0.001)	-5.0	-4.42343*10^-3^	Inf.^a^	4
			-5.0	-2.94895*10^-3^	2	5
			0	-7.62940*10^-7^	2	
			-5.0	-1.31268*10^-3^	2	6
			+5.0	1.18708*10^-3^	2	
Morphology and structure versus voltage amplitude	CdS (0.001)	Na_2_S (0.001)	-2.5	-5.81360*10^-4^	2	7
			+2.5	2.34985*10^-4^	2	
			-5.0	-1.30234*10^-3^	2	8
			+5.0	1.20498*10^-3^	2	
			-6.0	-2.07529*10^-3^	2	9
			+6.0	1.54178*10^-3^	2	
			-7.0	-3.50537*10^-3^	2	10
			+7.0	2.50537*10^-3^	2	
			-9.0	-5.00245*10^-3^	2	11
			+9.0	3.23547*10^-3^	2	
			-10.0	-6.65894*10^-3^	2	12
			+10.0	5.85234*10^-3^	2	
Different concentration with voltage modulation	CdS (0.003)	Na_2_S (0.003)	-2.5	-2.36574*10^-3^	2	13
			+2.5	1.78911*10^-3^	2	
			-5.0	-3.95514*10^-3^	2	14
			+5.0	4.87913*10^-3^	2	
			-6.0	-6.93433*10^-3^	2	15
			+6.0	4.98143*10^-3^	2	
			-7.0	-7.68741*10^-3^	2	16
			+7.0	5.35164*10^-3^	2	
			-9.0	-8.09984*10^-3^	2	17
			+9.0	6.15614*10^-3^	2	
			-10.0	-9.88776*10^-3^	2	18
			+10.0	8.00364*10^-3^	2	
	CdS (0.003)	Na_2_S (0.005)	-2.5	-4.06871*10^-3^	2	19
			+2.5	3.99878*10^-3^	2	
			-5.0	-6.79454*10^-3^	2	20
			+5.0	5.51788*10^-3^	2	
			-6.0	-8.23256*10^-3^	2	21
			+6.0	6.84196*10^-3^	2	
			-7.0	-9.94613*10^-3^	2	22
			+7.0	9.21516*10^-3^	2	
			-9.0	-1.36516*10^-2^	2	23
			+9.0	1.24556*10^-2^	2	
			-10.0	-2.31949*10^-2^	2	24
			+10.0	1.93546*10^-2^	2	

^a^“Inf.” in the voltage column means potentiostatic condition.

Generally, the results proved that the effective growth of CdS can take place on the inner surface of the TiO_2_ NTAs. In this system, multiple procedures are involved due to possible reaction paths of ionic S_2_O_3_^2-^ anions [31] with other species, which makes it more complicated for the attempt to control the reaction. Furthermore, though assisted by electric field, the reaction rate is still in the time scale of hours. Simply reducing the reaction time only led to less deposition near the open end of the tubes but in the meantime even less inside the tubes. Besides, pH control is also important here since the material quality can be influenced even by a small change in pH value [30,47]. Therefore, we have firstly replaced the precursors with Na_2_S and Cd(NO_3_)_2_ instead of Na_2_S_2_O_3_ and CdCl_2_, in order to accelerate the releasing of S^2-^ and the process of CdS formation with the introduction of the NO^3-^ anions [31,48]. To prevent the formation of CdS in the solution due to the fast reaction between Na_2_S and Cd(NO_3_)_2_, Cd(NO_3_)_2_ was very slowly injected by a syringe connected with the electrolyzing cell, and the voltage was set higher. To further simplify the process, the reaction was in neutral solution and under RT.

**Figure 2:**
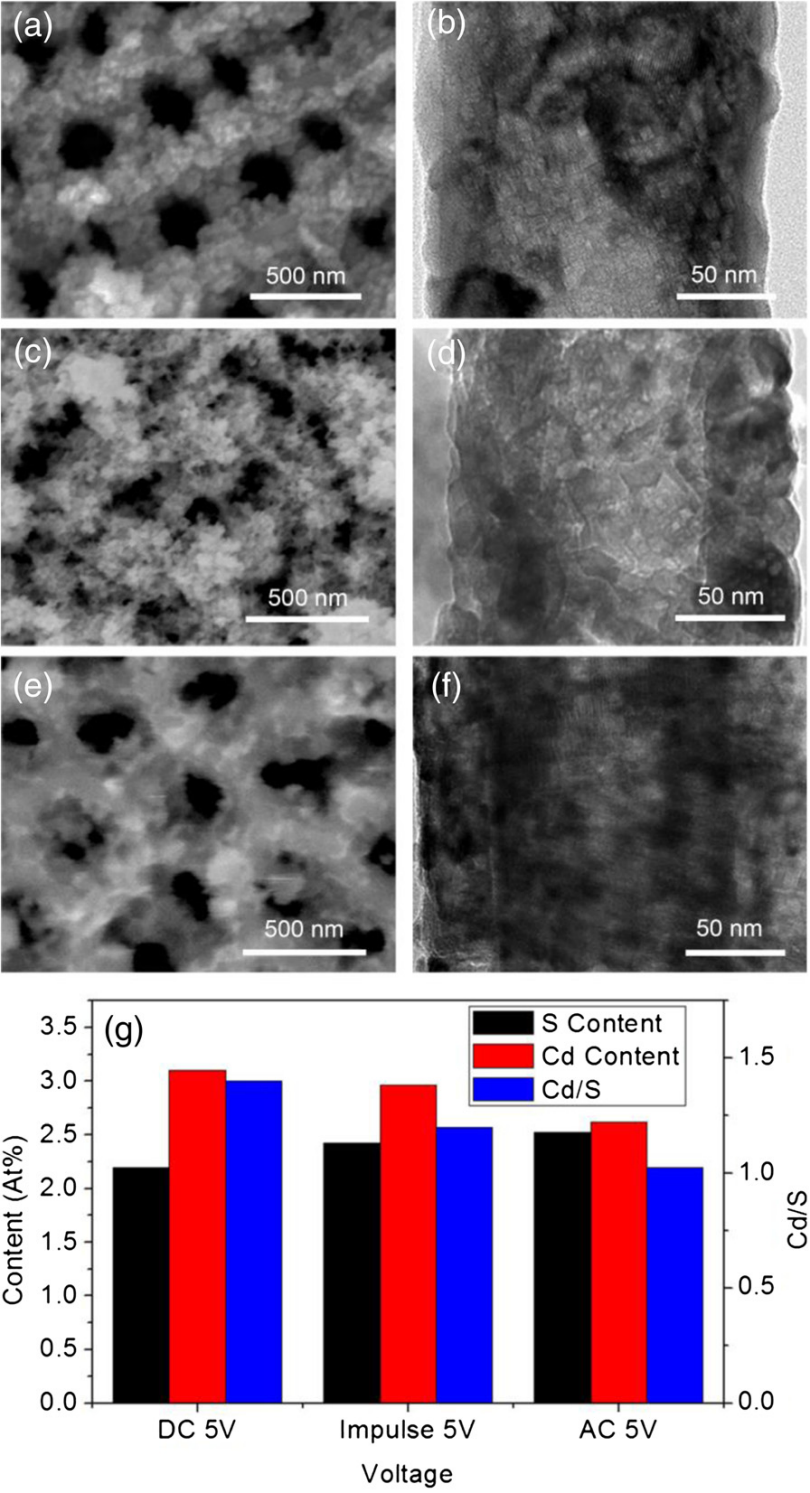
**CdS deposition under different applied voltages. (a-f)** Samples under different types of voltage: **(a) (b)** constant voltage, -5.0 V, SEM (top view) and TEM (lateral). **(c) (d)** impulse voltage, -5.0 V, 0.25 Hz, SEM (top view) and TEM (lateral). **(e) (f)** AC voltage, ±5.0 V, 0.25 Hz, SEM (top view) and TEM (lateral). **(g)** The content and ratio of Cd and S versus different voltages, with a growth time of 1 h.

In the new system, as shown in Figure 2a,b, small grains with the size of about 5 to 6 nm were formed inside and outside the TiO_2_ NTAs at bias of -5.0 V (the detailed conditions of deposition can be found in Table 1). Though the blocking effect is less compared to the situation of Figure 1c,d, the amount of the deposited material at the open end of the NTAs was still significantly much more than that inside the tubes. This has been comprehended to be related to the repulsing effect of the negative bias on the substrate to the reacting anions (here is S^2-^). To try to reduce that, we have firstly tried to apply impulse voltage by which the anions have more chance to move into the tubes by diffusion between two negative pulses. As shown in Figure 2c,d, with the same voltage magnitude, unlike the significant grains in potentiostatic condition, the deposited material at the top of NTAs became much more deformed, while it tended to form larger grains inside the tubes, though the top surface of NTAs was still covered by a large amount of deposited material. Hence, alternative voltage (±5.0 V, square wave) was applied, which would hopefully offer extra drifting force for the motion of anions (S^2-^) toward reaction site in the tubes. Due to the fast reaction between Cd^2+^ and S^2-^, the repulsing effect of the positive half-wave on the Cd^2+^ can be little. As illustrated by Figure 2e,f, a significantly uniform thin film has been formed inside the TiO_2_ NTAs, though the surface at the open end of the NTAs was still covered by a certain amount of deposited material. Further analysis was done by EDX measurement, in which the amount of Cd and S was detected on the same sample. The signal intensity of Cd and S was measured and compared by the ratio between them, as demonstrated in Figure 2g. Under constant bias at -5.0 V, the contents of Cd and S on the sample surface were 3.1% and 2.2%, respectively, with a molar ratio of 1.4:1. When impulse voltage was applied, the Cd and S contents are 2.96% and 2.43%, respectively, with a molar ratio of 1.2:1. This is likely due to the diffusion process of S in between each pulse. Further experiment on AC voltage has shown that this tendency can be more enhanced. As shown in the third group of data in Figure 2g, the content of Cd and S were reduced and increased, respectively, so that their ratio has reached approximately 1 to 1.03. Combined with the morphological studies in Figure 2a,b,c,d,e,f, it can be concluded that the alternative voltage can be suitable to grow CdS thin film with good quality inside the TiO_2_ NTAs.

**Figure 3:**
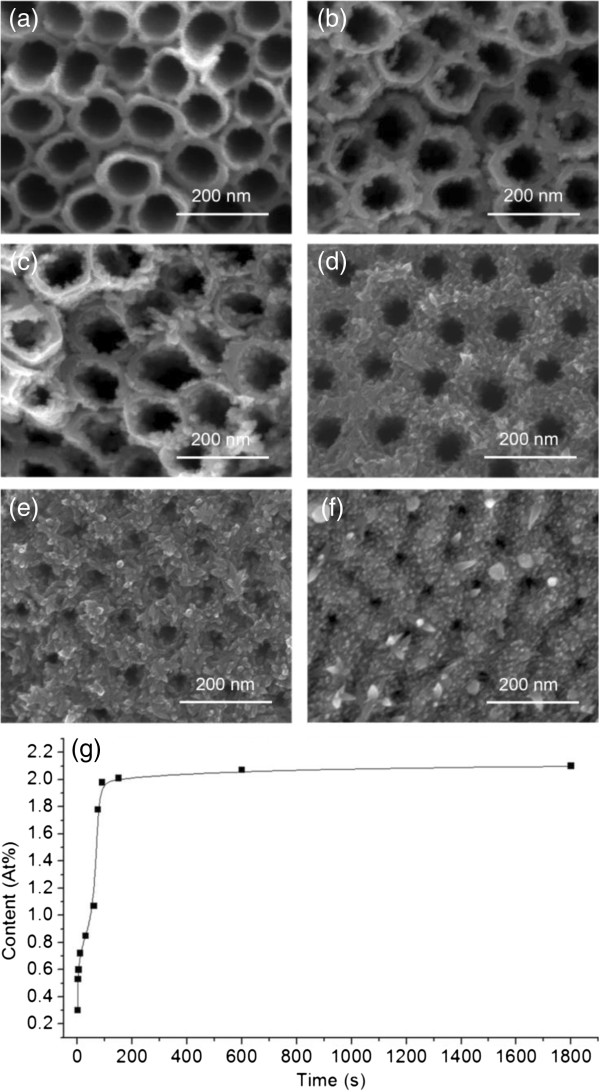
**Rapid growth of CdS into TiO**_**2 **_**NTAs. (a-d)** SEM image (top view) of CdS-coated TiO_2_ NTAs under different reaction times: **(a) ***t* = 1 s, **(b) ***t* = 60 s, **(c) ***t* = 75 s, **(d) ***t* = 90 s, **(e) ***t* = 600 s, and **(f) ***t* = 1,800 s. **(g)** Growth curve of CdS content based on EDX data versus different reaction times.

Furthermore, during the previous reactions with an electric field assistance of a 1-h reaction period, it is noteworthy that there was significant color changing soon after the experiments started, and it remained almost unchanged during the rest of the time. It indicates that the real deposition may have lasted in a much shorter period. To test that, we carried out experiments of the CdS formation versus time under AC voltages with equally heighted cathodic and anodic pulses. Results and detailed conditions can be found in Figure 3 and Table 1, respectively. Figure 3a,b,c,d,e,f is the SEM images of the samples formed at different time points, and Figure 3g is the EDX intensity versus time. According to the result shown in Figure 2, under AC voltage condition (±5 V), the content of Cd was used to represent the amount of the CdS deposition (by EDX signal). In the beginning, the growth mainly took place inside the tubes (when *t* < 60 s, as shown in Figure 3a,b). After 60 s, the growth started at the top surface and continued to cover it, as shown in Figure 3c,d,f. The top surface of the NTAs was almost entirely covered by CdS at about 500 s. In the meantime, the EDX measurement in Figure 3g has shown that the content of CdS rapidly increased in the beginning, until it reached the value of 2.0% at 90 s. Afterwards, the increase of the general amount of CdS significantly slowed down, although the surface of the top of NTAs was still continuously covered by CdS. Therefore, we can draw two conclusions here: 1) The reaction took place very quickly in the beginning 90 s; 2) fast growth of CdS stopped at about 90 s, and after then, the surface opening continue to reduce significantly, but the content of CdS increased very slowly. This suggests that effective CdS growth achieved two magnitudes faster than the previous works [22,27]. Furthermore, it suggests too long growth time may not be suitable for the application requiring adequate surface openings.

**Figure 4:**
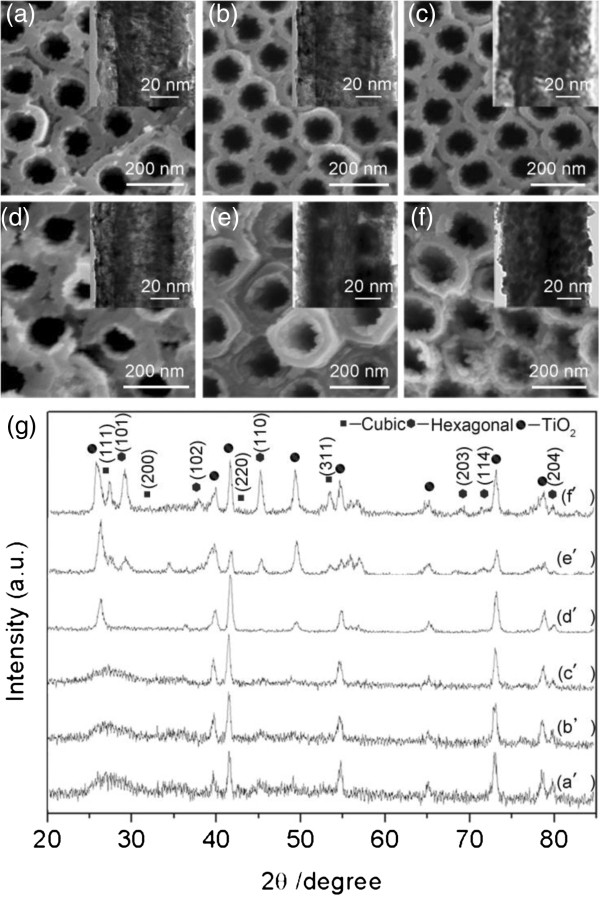
**Evolution of morphology and microstructure of CdS deposition in TiO**_**2 **_**NTAs under AC conditions. (a-f)** SEM images (top view) of CdS-coated TiO_2_ NTAs (insets are corresponding TEM images) with different bias magnitudes: **(a)** |*U*| = 2.5 V, **(b)** |*U*| = 5.0 V, **(c)** |*U*| = 6.0 V, **(d)** |*U*| = 7.0 V, **(e)** |*U*| = 9.0 V, and **(f)** |*U*| = 10.0 V. **(g)** XRD patterns, with curves (a’-f’) for corresponding samples, concentration of Na_2_S was 0.001 M. Detailed conditions of deposition can be found in Table 1.

In previous experiments, we have found that CdS nanomaterial can be successfully grown into TiO_2_ NTAs in a very short time scale with the application of AC voltage-assisted chemical deposition. In TiO_2_ NTAs, it is still unknown how the voltage magnitude would influence the as-grown material. Therefore, we have carried out experiments under AC voltage with different absolute magnitudes. Figure 4a,b,c,d,e,f has presented results with the magnitude (all with equal height in cathodic and anodic pulses) from 2.5 to 10.0 V. At a magnitude of 2.5 V, a significant continuous film was grown inside the tubes, while only little deposition as island formation can be observed at the tube openings, and it remained clean on top of the NTAs. This has significantly differed from the previous results shown in Figure 1c using a conventional method. The average thickness was about 15 nm at 2.5 V. As the magnitude of voltage increased to 5.0 and 7.0 V, the continuous film became slightly thicker inside the tubes, while more islands developed at the inner surface of the TiO_2_ NTAs. The upper end of the tube arrays remained open, and no top cover appeared above the NTAs, although more islands have formed there. When a magnitude of the voltage rose to 9.0 V, the film inside the tubes became significantly thicker (~24 nm) while the islands became stuck together at the upper end of the tubes. Finally, at 10.0 V, the tubes were almost totally filled with deposited materials, and certain cover began to appear above the top of the tubes. It can be concluded that as the magnitude of the field intensity increased, more deposition has taken place in the inner surface of the tubes; in the meantime, significant deposition on top of the tube openings (as shown in Figure 1c,d) was avoided.

Moreover, we have noticed a certain color change with the voltage increase during the whole process, that the color of the sample gradually changed from lemon yellow (2.5 and 5.0 V), to jacinth (6.0, 7.0, and 9.0 V), and finally to light black (10.0 V) (corresponding pictures of samples can be found in Additional file 1: Figure S3). As we know, yellow and red are typical colors of the α-hexagonal and β-cubic phase CdS crystalline structures, respectively. Therefore, this result has implied that certain structural change might have taken place with the increase of voltage magnitude. To verify that, we characterized the samples with XRD measurement, with the results shown in Figure 4g. Curves a’-f’ are XRDs of samples under corresponding conditions presented in Figure 4a,b,c,d,e,f, respectively. The JPDS 41-1049 Hexagonal (H) and JCPDS 10-0454 Cubic (C) reference patterns are used to identify the observed diffraction patterns. With a magnitude of voltage of 2.5 V, the XRD of the sample has characteristic peaks at 44.6° and 77.8° that are corresponding to (110) and (204) face of hexagonal structure of CdS, respectively. When the magnitude of voltage rose to 5.0 and 6.0 V, the XRD peaks were similar. At 7.0 V, a weak peak at 35.5° appeared, which is corresponding to (102) of the hexagonal structure. At 9.0 V, a peak appeared at 26.8° and 52.3°, indicating (111) and (311) faces of the cubic structure of CdS, respectively. When the voltage magnitude was further increased to 10.0 V, the peak at 26.8° became stronger. In the meantime, the peaks at 55.5° in curves e’ and f’ indicate the possible existence of CdO material under 9.0 and 10.0 V [49], which was further supported by EDX measurement (the more quantitative estimation is given in Additional file 1: Table S2) and may partly explain the color change at 9.0 and 10.0 V. Apparently, this result suggested certain transformation of the crystallization structure of the as-deposited CdS material as the voltage magnitude increased. What’s more, with higher voltage magnitude, the CdS nanomaterial tends to form a rigid film rather than simply packed CdS particles at the inner surface of NTAs.

**Figure 5:**
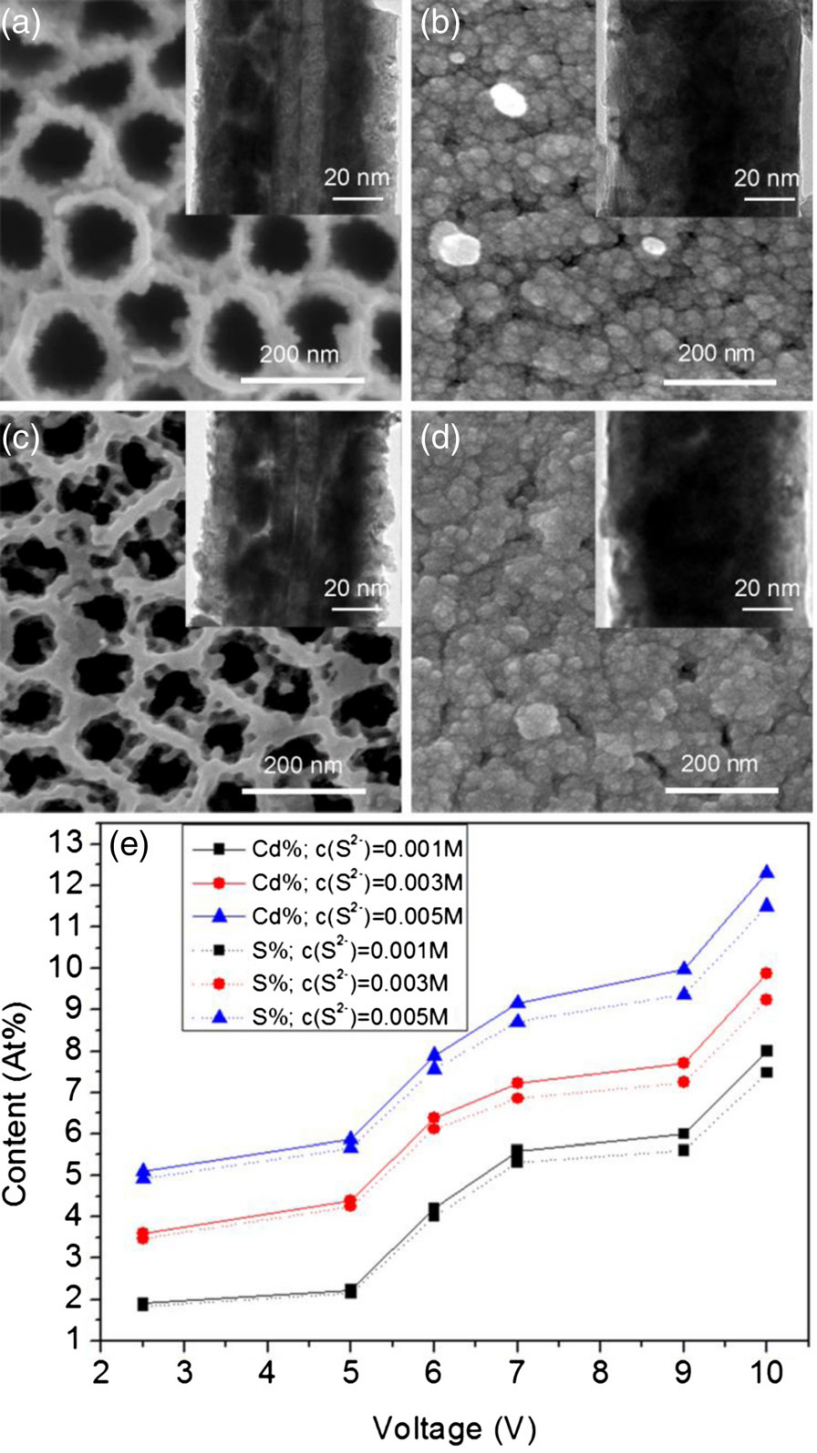
**Influence of precursor concentration on the formation of CdS. (a) (b)** SEM and TEM images of samples with precursor concentration (Na_2_S and Cd(NO_3_)_2_) of 0.003 M, at voltage magnitudes of **(a)** 7.0 V and **(b)** 9.0 V, respectively. **(c) (d)** SEM and TEM images of samples with precursor concentration (Na_2_S and Cd(NO_3_)_2_) of 0.005 M; at voltage magnitudes of **(c)** 7.0 V and **(d)** 9.0 V, respectively. **(e)** Growth curve of as-deposited CdS content based on EDX data versus different applied bias voltage and different concentration of precursors. Detailed conditions of deposition can be found in Table 1.

Till now, we can see that tuning the applied AC voltage in the chemical deposition system can effectively change not only the chemical content of the as-deposited materials in the NTAs but also their microstructures. Furthermore, with the increasing voltage magnitude, the thickness of the deposited inner layer continued to increase as well (Figure 4). Apparently, it contributes to a rigid inner layer inside the TiO_2_ nanotubes. However, when voltage was too high, the as-formed film would turn to black color; other contents like CdO appeared to form, i.e., simply increasing voltage to enhance the deposition has a certain limitation. Therefore, we applied a similar experiment with different precursor (Na_2_S/Cd(NO_3_)_2_ = 1:1) concentrations at 0.003 and 0.005 M. The comparison of interested conditions is shown in Figure 5 for samples under a bias magnitude of 7.0 and 9.0 V. Under 0.003 M concentration, the tubes can be identified with thicker films at 7.0 V than that under 0.001 M and became filled up at 9.0 V. Moreover, the top of NTAs was almost blocked by a thick cover, with only small holes left. Under 0.005 M concentration, the tubes tend to be filled up already at 7.0 V, and at a magnitude of 9.0 V, the top of them was totally covered.

To get more quantitative information about the content variation versus the voltage magnitude and precursor concentration, EDX measurements were carried out for all samples, as shown in Figure 5e. First of all, the contents of Cd and S were very close in the whole experimental range (with the detailed value shown in Additional file 1: Table S3). It has further proved that AC voltage has a significant contribution to form the CdS material with a quite high purity in this electric-field-assisted chemical deposition. This enables us to simplify the discussion of the amount of as-formed CdS with the Cd signal intensity. Secondly, the amount of as-formed CdS increased monotonously with increasing voltage under the same precursor concentration. It also significantly increased with higher precursor concentration at the same voltage magnitude. This is more obvious in the low voltage region (2.5 to 5.0 V). The ratio of the amount of as-formed CdS among different concentrations was close to 1:3:5, which was the ratio of the precursor concentrations. Finally, the Cd/S ratio was almost independent on the increasing voltage. Hence, the thickness of the CdS film can be effectively controlled by the precursor concentration and voltage magnitude (detailed relationship can be found in Additional file 1: Figure S4). Consider the CdS thickness, opening of the as-formed structures, and continuity of the film, a moderate experiment condition (6.0 to 7.0 V, 0.003 M precursor concentration) was seemly a suitable condition for application.

Previous results have revealed a significant fast growth of CdS material in a few minutes with controlled applied AC voltage and without any surfactant at a low temperature (20°C). The results can be attributed from several points. First, it is well known that Na_2_S and Cd(NO_3_)_2_ react almost immediately when they coexist in solution:

(1) $${Cd}^{2+}+S^{2-}\to CdS$$

It significantly differs from the conventional method by Na_2_S_2_O_3_ and CdCl_2_, which has normally the following major processes [50]:

(2) $$S_{2}{O_{3}}^{2-}+H^{+}\to S+H_{2}{\mathrm{SO}}_{3}+2H_{2}O$$

(3) $$S+2H^{+}+2e^{-}\to H_{2}S$$

(4) $$H_{2}S+{Cd}^{2+}\to CdS+2H^{+}$$

This enables the very fast development of CdS formation. It is sure that simply introducing them simultaneously would result to the formation of CdS particles in the solution even at a concentration much lower than the lowest value applied in this experiment. Moreover, the distribution of as-deposited material would be higher near the tube opening but less in the inside due to the influence of the concentration gradient on the reaction of the ions (Cd^2+^ and S^2-^ in this system) in the tubes.

If taking a closer look at the reaction in a deposition system, the ionic reactions at any substrate would normally contains following procedures: the diffusion of dissolved species in the solution, reaction of ionic species in the solution (possible drifting effect by electric or mechanical force), adsorption of ions at the substrate, and reaction of them with each other. According to our results, suitably introduced electric field would significantly enhance the drifting process and, thus, the CdS formation at the substrate. Because Cd(NO_3_)_2_ was injected into the system with a very small flux (1 ml/s) compared to the amount of the Na_2_S (200 ml), the enhancement of the reaction at the substrate would have effectively weaken the reaction in the solution, and almost a clean solution can be left even after the whole deposition was accomplished.

What’s more, though the application of DC electric field can increase the distribution of deposited material inside the tubes, the ratio of Cd and S content in the as-fabricated can be far from 1:1, and thus, the purity of CdS was actually small due to a repulsing effect on anions while attracting the cations. This cannot be simply compensated by changing the ratio of Cd and S precursors without influencing the other factors (e.g., the voltage magnitude). This problem was resolved by the introduction of AC voltage (square wave). The independence of the Cd:S ratio (which means high content of CdS in the as-fabricated materials) with the voltage magnitude can be due to the faster reaction of the adsorbed Cd^2+^ and S^2-^ ions near the tube inner surface compared to their motion in- and outward the tubes.

Furthermore, the as-fabricated material has certain structure dependence on the voltage magnitude. According to Jun-Heng Xing’s result, electric field can offer extra energy for the activation energy of crystallization process [51]. For α-hexagonal and β-cubic, its bond energy are 229.4 and 187.6 D°_298_/kJ mol^-1^, respectively [52]. Therefore, if gradually increasing the external voltage, crystalline structures related to hexagonal and cubic can appear in sequence. However, this process might not be complete due to the randomization of the current, and ordering in certain directions could be missing. This could hopefully be improved by varying the external energy with the manipulation of other conditions (light field) or the aid of post treatment.

**Figure 6:**
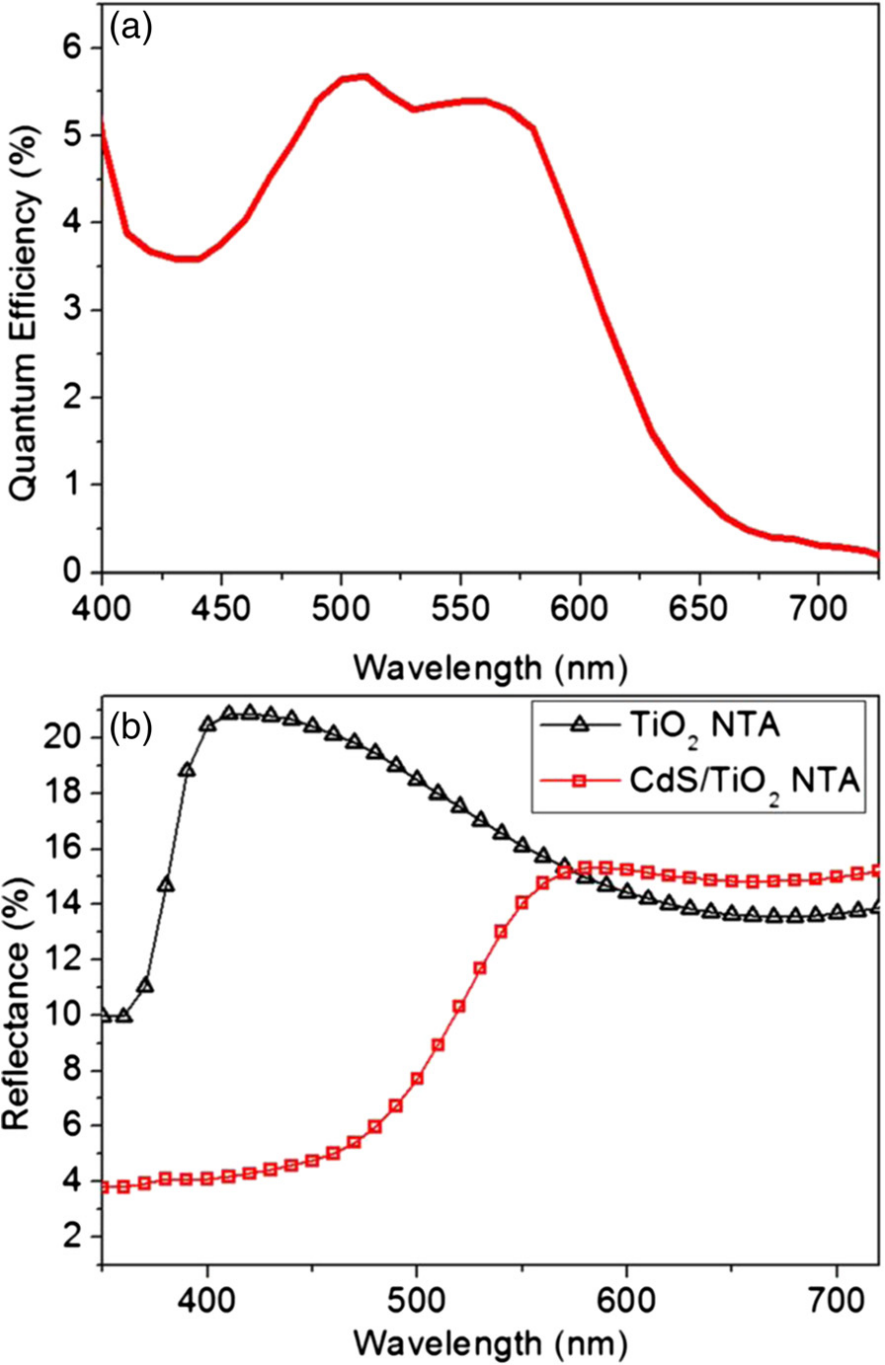
**Optoelectronic properties of as-fabricated CTF/TNT structure-based solar cell (precursor concentration 0.003 M, voltage magnitude 7.0 V). (a)** Quantum Efficiency. **(b)** Reflectance spectrum.

Finally, the as-fabricated material was integrated into TiO_2_ NTAs and form sensitized solar cell (back-side illumined) to make further characterizations. Figure 6a shows the quantum efficiency of the as-fabricated device. According to previous discussions, the deposition was carried out with a precursor concentration of 0.003 M and voltage magnitude of 7.0 V. Different from typical quantum dots, the sample has shown a broad peak between 500 and 600 nm, corresponding to photon energy from 2.48 to 2.07 eV. For reference, the band gap of CdS bulk is 2.4 eV, corresponding to the wavelength of about 518 nm, while this value was measured to about 2.8 eV for CdS quantum dots sized 5 ~ 7 nm, corresponding to wavelength of about 443 nm by other groups [53]. This result has shown a similar behavior of deposited CdS by SILAR method in the work of other groups [53], whose origin was related to a high degree of disorder in the CdS film and the nonuniform absorption of UV-vis light of different wavelengths within the film [2]. The broad absorption from 500 to 600 nm would hopefully result better light absorption and carrier excitation for the photon energy lower than the 2.4 eV. It is further supported by the comparison of the reflection spectra between the deposited sample and bare TiO_2_ NTAs.

Considering the shape and range of AM1.5 spectrum [54], this characteristic quantum efficiency curve of this sample has a potential advantage for matching the solar spectrum.

**Figure 7:**
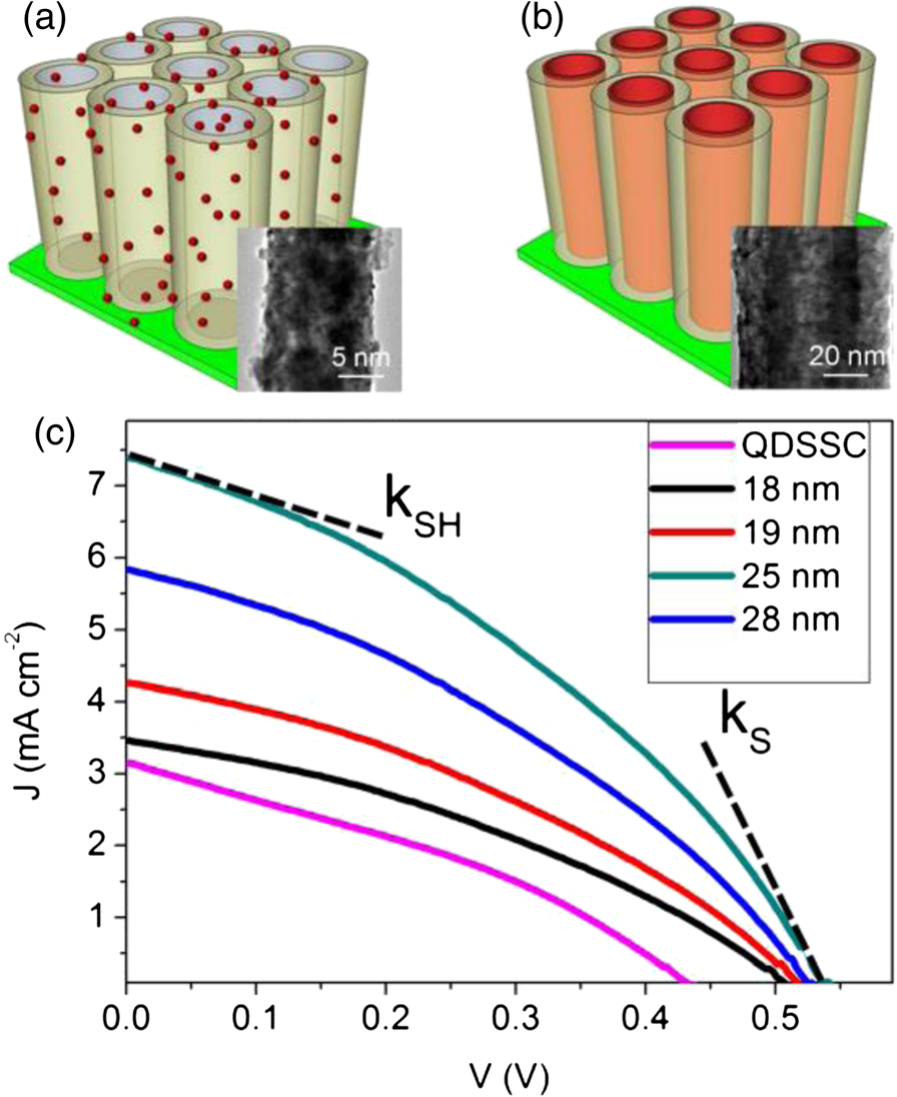
**Configuration and characteristics of CdS QDSSC and thin film solar cell. (a)** Structure diagram of QDSSC and the inset is the TEM image of QDs in TiO_2_ NTAs. **(b)** Structure diagram of thin film solar cell and the inset is the TEM image of the complex tubular structure. **(c)** J-V characteristics of sensitized solar cells based on CdS QDs and thin films of different thicknesses.

To further investigate the performance of those CTF/TNT structures in the sensitized cells, CdS films with different thicknesses were fabricated at a voltage from 2.5 to 10.0 V with a precursor concentration of 0.003 M. Furthermore, J-V characteristics of the cells have been measured together with QDSSC fabricated with the same method (QDs of size about 3 to 7 nm were fabricated in situ in the NTAs by simply shortening the growth time to 40 s, as shown in Figure 7a). Firstly, all cells with CdS films had significantly higher *J*_SC_, *V*_OC_, and *η* than the QDSSC. Secondly, as shown in Figure 7c, the *J*_SC_, *V*_OC_, and *η* first increased with increasing CdS film thickness and then began to drop when thickness was higher than 25 nm (with detailed values given in Additional file 1: Table S4). The cell with CdS thickness of 25 nm had the highest *η* of 1.43% (with *J*_SC_ = 7.40 mA cm^-2^, *V*_OC_ = 0.55 V, and FF = 0.35), 210% higher than the value of QDSSC with a similar condition (*η* = 0.46%, *J*_SC_ = 3.15 mA cm^-2^, *V*_OC_ = 0.44 V, and FF = 0.33).

It could be expected that the initial increase of the cell performance with the thickness increase below 25 nm can be related to more photo-induced carrier generation by the more amount of CdS or thicker CTF in this experiment. However, the decrease of *η* and *J*_SC_ for a thickness above 25 nm cannot simply be explained by this. Therefore, we have to look more deeply into some cell properties concerning the carrier transport in a CdS layer. We have measured the absolute values of the slopes near *J*_SC_ and *V*_OC_ (*k*_SH_ and *k*_S_, indicated by dashed tangents in Figure 7), which are proportional to the series and shunt resistances, respectively [55]. On the one hand, *k*_*S*_ was 1.29 × 10^-2^ Ω^-1^ cm^-2^ for the QDSSC. For the CdS thin film-sensitized cell, this value was 1.35, 1.71, 2.99, and 2.28 × 10^-2^ Ω^-1^ cm^-2^ for the film thickness 18 (2.5 V), 19 (5.0 V), 25 (7.0 V), and 28 nm (9.0 V), respectively. Apparently, the series resistance of the cell based on CdS film was much lower than that of the simple QDSSC, which was likely due to a larger contact area than that of the QDs. Moreover, interestingly, there was an initial decrease of this value when film thickness increased from 18 to 25 nm, on the contrary to normal positively proportional relationship of material series resistance and its thickness (which would be detected later for thickness higher than 25 nm). It might be due to the better crystallization of the deposited CdS at a higher voltage if thickness was not too high. After 25 nm (corresponding to the voltage magnitude of 7.0 V), the influence from this factor was weak, and the normal rule again dominant the whole process.

On the other hand, *k*_SH_ of QDSSC was about 5.29 × 10^-3^ Ω^-1^ cm^-2^. Of the thin CdS film-sensitized cell was 4.16, 4.15, 4.10, and 4.38 × 10^-3^ Ω^-1^ cm^-2^ for the film thickness 18, 19, 25, and 28 nm, respectively. Compared to QDSSC, the thin film-sensitized cell had generally less shunt resistance, which might be due to its lower probability of recombination with better electron transport within the CTF structure. However, when film thickness increased, the shunt resistance would firstly increase and then decrease (thickness >25 nm) again. Similar to the discussion on series resistance, the initial increase of shunt resistance would have been resulted from the less recombination of electrons due to the better crystallization of deposited film with a higher voltage magnitude. While for higher thickness (>25 nm), the influence of the material thickness seemed to be a dominant factor that increased the chance for the recombination of carriers during their transport in a thicker film. Generally speaking, this behavior is consistent with the previously described nonmonotonous evolution of J-V characteristics, and we can hereby conclude that a medium thickness would be the optimum condition that balances carrier generation and transport processes. In future development, the cell performance could still be improved with alternations such as the application of more suitable electrolyte/counter electrode system and the flux control of the precursors by mass flow controllers.

Beside photovoltaic devices, the broad band distribution of the CTF/TNT structure can also be useful for broad-spectrum light emission [56]. It would also effectively suppress the retard of the electron flow and reduce the thickness for super-capacitors [57], and have high sensitivity, good temporal, and spatial control in electrochemiluminescence (ECL) detection of biological recognition [58]. It is also noticeable that the whole fabrication process has taken place in an open system if considering the similar time scale of the precursor injection (100 s) and reaction period (1 to 2 min). With potential modifications (i.e., continuous precursor injection by micropumps and control by mass flow controller) of the growth method, more effective designing and manipulation of nanostructure formation process would hopefully be expected.

## Conclusions

In general, this experiment has investigated a composite CdS/TiO_2_ structure (TiO_2_ nanotubes with homogeneous CdS coating inside) by a new ultrafast growth method with the growth time (~90 s) two orders of magnitude shorter than similar methods [27] at RT. The thickness of the as-formed film inside the tubes can be monotonously tuned by the voltage magnitude and precursor concentrations. The quality of deposition like the content ratio can be greatly improved by suitable voltage control. Furthermore, the crystalline structures of the as-formed CdS can be changed versus the different magnitude of voltage. The sensitized solar cells composed of as-fabricated complex structures on TiO_2_ NTA substrate have quite a wide distribution of energy band gap, which indicates potential possibility in spectrum matching with band manipulation. As a result, the as-formed sample has shown a significant advantage than conventional structures in several aspects. We name the growth method “successive electrochemical deposition (SECD)” since its reactions took place in an open system. This work would not only be useful for CdS into TiO_2_ NTAs but also the trend to rapidly grow other material into different nanostructures and for a wide range of device applications.

## Abbreviations

ALD: Atomic layer deposition; CTF: CdS thin film; CBD: Chemical bath deposition; DSSCs: Dye-sensitized solar cells; ECD: Electrochemical deposition; EDX: Energy dispersive X-ray; FE-SEM: Field emission scanning electron microscopy; FF: Fill factor; NP: Nanoporous; NTAs: Nanotube arrays; QD: Quantum dot; QDSSCs: Quantum dot-sensitized solar cells; QE: Quantum efficiency; RT: Room temperature; SECD: Successive electrochemical deposition; SILAR: Successive ionic layer adsorption and reaction; TNT: TiO_2_ nanotube; TEM: Transmission electron microscopy.

## Competing interests

The authors declare that they have no competing interests.

## Authors’ contributions

HL raised the initial idea and was in charge of the experiment design, paper design, and correction. HF carried out the experiments, data analysis, and writing of the manuscript. WS gave valuable advice in the discussions on the experiment and the paper structures and was in charge of the verification on the final form of the paper. All authors read and approved the final manuscript.

## Supplementary Material

Additional file 1 **Supporting information.** This file contains the schematic diagram of the device, the waveform of the applied voltage, the pictures of outcome materials, the relationship of film thickness and growth conditions, and the EDX data on material contents under different conditions, and J-V characteristics of as-fabricated back-side-illuminated solar cells. Click here for file

## References

[B1] Xie Y Ali G Yoo SH Cho SO Sonication-assisted synthesis of CdS quantum-dot-sensitized TiO_2_ nanotube arrays with enhanced photoelectrochemical and photocatalytic activity Acs Appl Mater Inter 2010 2 2910 2914 10.1021/am100605a 20849087

[B2] Baker DR Kamat PV Photosensitization of TiO_2_ nanostructures with CdS quantum dots: particulate versus tubular support architectures Adv Funct Mater 2009 19 805 811 10.1002/adfm.200801173

[B3] Wang CL Sun L Yun H Li J Lai YK Lin CJ Sonoelectrochemical synthesis of highly photoelectrochemically active TiO_2_ nanotubes by incorporating CdS nanoparticles Nanotechnology 2009 20 1361 6528 10.1088/0957-4484/20/29/295601 19567967

[B4] Shin K Il Seok S Im SH Park JH CdS or CdSe decorated TiO_2_ nanotube arrays from spray pyrolysis deposition: use in photoelectrochemical cells Chem Commun 2010 46 2385 2387 10.1039/b923022j 20379539

[B5] Kongkanand A Tvrdy K Takechi K Kuno M Kamat PV Quantum dot solar cells. tuning photoresponse through size and shape control of CdSe-TiO2 architecture J Am Chem Soc 2008 130 4007 4015 10.1021/ja0782706 18311974

[B6] Zhang H Quan X Chen S Yu HT Ma N “Mulberry-like” CdSe nanoclusters anchored on TiO_2_ nanotube arrays: a novel architecture with remarkable photoelectrochemical performance Chem Mater 2009 21 3090 3095 10.1021/cm900100k

[B7] Gao XF Li HB Sun WT Chen Q Tang FQ Peng LM CdTe quantum dots-sensitized TiO_2_ nanotube array photoelectrodes J Phys Chem C 2009 113 7531 7535 10.1021/jp810727n

[B8] Seabold JA Shankar K Wilke RHT Paulose M Varghese OK Grimes CA Choi KS Photoelectrochemical properties of heterojunction CdTe/TiO_2_ electrodes constructed using highly ordered TiO_2_ nanotube arrays Chem Mater 2008 20 5266 5273 10.1021/cm8010666

[B9] Plass R Pelet S Krueger J Gratzel M Bach U Quantum dot sensitization of organic-inorganic hybrid solar cells J Phys Chem B 2002 106 7578 7580 10.1021/jp020453l

[B10] Liu B Aydil ES Growth of oriented single-crystalline rutile TiO_2_ nanorods on transparent conducting substrates for dye-sensitized solar cells J Am Chem Soc 2009 131 3985 3990 10.1021/ja8078972 19245201

[B11] Hochbaum AI Chen RK Delgado RD Liang WJ Garnett EC Najarian M Majumdar A Yang PD Enhanced thermoelectric performance of rough silicon nanowires Nature 2008 451 163 U5 10.1038/nature06381 18185582

[B12] Tian BZ Zheng XL Kempa TJ Fang Y Yu NF Yu GH Huang JL Lieber CM Coaxial silicon nanowires as solar cells and nanoelectronic power sources Nature 2007 449 885 U8 10.1038/nature06181 17943126

[B13] Huynh WU Dittmer JJ Alivisatos AP Hybrid nanorod-polymer solar cells Science 2002 295 2425 2427 10.1126/science.1069156 11923531

[B14] Foong TR Shen Y Hu X Sellinger A Template‒directed liquid ALD growth of TiO_2_ nanotube arrays: properties and potential in photovoltaic devices Adv Funct Mater 2010 20 1390 1396 10.1002/adfm.200902063

[B15] Bu YY Chen ZY Li WB Yu JQ High-efficiency photoelectrochemical properties by a highly crystalline CdS-sensitized ZnO nanorod array Acs Appl Mater Inter 2013 5 5097 5104 10.1021/am400964c 23688263

[B16] Su CK Sun YC Chemically differentiating ascorbate-mediated dissolution of quantum dots in cell culture media Nanoscale 2013 5 2073 2079 10.1039/c2nr33365a 23377100

[B17] Feng M Zhan HB Miao L Facile assembly of cadmium sulfide quantum dots on titanate nanobelts for enhanced nonlinear optical properties Acs Appl Mater Inter 2010 2 1129 1135 10.1021/am100003p 20423131

[B18] Grätzel M Recent advances in sensitized mesoscopic solar cells Accounts Chem Res 2009 42 1788 1798 10.1021/ar900141y 19715294

[B19] Li LB Wang YF Rao HS Wu WQ Li KN Su CY Kuang DB Hierarchical macroporous Zn_2_SnO_4_-ZnO nanorod composite photoelectrodes for efficient CdS/CdSe quantum dot co-sensitized solar cells Acs Appl Mater Inter 2013 5 11865 11871 10.1021/am4035653 24191709

[B20] Bae WK Padilha LA Park YS McDaniel H Robel I Pietryga JM Klimov VI Controlled alloying of the core-shell interface in CdSe/CdS quantum dots for suppression of auger recombination ACS Nano 2013 7 3411 3419 10.1021/nn4002825 23521208

[B21] Brennan TP Ardalan P Lee HBR Bakke JR Ding IK McGehee MD Bent SF Atomic layer deposition of CdS quantum dots for solid-state quantum dot sensitized solar cells Adv Energy Mater 2011 1 1169 1175 10.1002/aenm.201100363

[B22] Zhu W Liu X Liu HQ Tong DL Yang JY Peng JY Coaxial heterogeneous structure of TiO_2_ nanotube arrays with CdS as a superthin coating synthesized via modified electrochemical atomic layer deposition J Am Chem Soc 2010 132 12619 12626 10.1021/ja1025112 20536235

[B23] Jeong DK Park NH Jung SH Jung WG Shin H Lee JG Kim JY Fabrication of oxide/semiconducting coaxial nanotubular materials using atomic layer deposition Mater Sci Forum 2004 449–452 1165 1168

[B24] Zyoud A Saa’deddin I Khudruj S Hawash ZM Park D Campet G Hilal HS CdS/FTO thin film electrodes deposited by chemical bath deposition and by electrochemical deposition: a comparative assessment of photo-electrochemical characteristics Solid State Sci 2013 18 83 90

[B25] Pawar SM Pawar BS Kim JH Joo OS Lokhande CD Recent status of chemical bath deposited metal chalcogenide and metal oxide thin films Curr Appl Phys 2011 11 117 161 10.1016/j.cap.2010.07.007

[B26] Ravichandran K Senthamilselvi V Effect of indium doping level on certain physical properties of CdS films deposited using an improved SILAR technique Appl Surf Sci 2013 270 439 444

[B27] Lee HJ Chen P Moon SJ Sauvage F Sivula K Bessho T Gamelin DR Comte P Zakeeruddin SM Seok SI Grätzel M Nazeeruddin MK Regenerative PbS and CdS quantum dot sensitized solar cells with a cobalt complex as hole mediator Langmuir 2009 25 7602 7608 10.1021/la900247r 19499942

[B28] Sun WT Yu Y Pan HY Gao XF Chen Q Peng LM CdS quantum dots sensitized TiO_2_ nanotube-array photoelectrodes J Am Chem Soc 2008 130 1124 1125 10.1021/ja0777741 18183979

[B29] Ilieva M Dimova-Malinovska D Ranguelov B Markov I High temperature electrodeposition of CdS thin films on conductive glass substrates J Phys-Condens Mat 1999 11 10025 10031 10.1088/0953-8984/11/49/320

[B30] Chassaing E Naghavi N Bouttemy M Bockelee V Vigneron J Etcheberry A Lincot D Electrodeposition mechanism of indium sulfide and indium oxi(hydroxi)sulfide thin films from In(III)-thiosulfate acidic aqueous solutions J Electrochem Soc 2012 159 D347 10.1149/2.051206jes

[B31] Izgorodin A Winther-Jensen O Winther-Jensen B MacFarlane DR CdS thin-film electrodeposition from a phosphonium ionic liquid Phys Chem Chem Phys 2009 11 8532 8537 10.1039/b906995j 19774284

[B32] Yong KT Sahoo Y Swihart MT Prasad PN Shape control of CdS nanocrystals in one-pot synthesis J Phys Chem C 2007 111 2447 2458 10.1021/jp066392z

[B33] Tang ZR Yin X Zhang Y Xu YJ Synthesis of titanate nanotube-CdS nanocomposites with enhanced visible light photocatalytic activity Inorg Chem 2013 52 11758 11766 10.1021/ic4010483 24074302

[B34] Abe T Kashiwaba Y Baba M Imai J Sasaki H XPS analysis of p-type Cu-doped CdS thin films Appl Surf Sci 2001 175 549 554

[B35] Albrasi E Thomas PJ O’Brien P Deposition of nanostructured films of CdSe and CdS using three layered water-oil-amphiphile/salt system J Mater Chem C 2013 1 671 676

[B36] Cheng X Pan G Yu X Zheng T Preparation of CdS NCs decorated TiO_2_ nano-tubes arrays photoelectrode and its enhanced photoelectrocatalytic performance and mechanism Electrochim Acta 2013 105 535 541

[B37] Chun S Han KS Lee JS Lim HJ Lee H Kim D Fabrication CdS thin film and nanostructure grown on transparent ITO electrode for solar cells Curr Appl Phys 2010 10 S196 S200 10.1016/j.cap.2009.07.030

[B38] Banerjee S Mohapatra SK Das PP Misra M Synthesis of coupled semiconductor by filling 1D TiO_2_ nanotubes with CdS Chem Mater 2008 20 6784 6791 10.1021/cm802282t

[B39] Chen C Ali G Yoo SH Kum JM Cho SO Improved conversion efficiency of CdS quantum dot-sensitized TiO_2_ nanotube-arrays using CuInS_2_ as a Co-sensitizer and an energy barrier layer J Mater Chem 2011 21 16430 16435 10.1039/c1jm13616j

[B40] Xiao FX Miao JW Wang HY Liu B Self-assembly of hierarchically ordered CdS quantum dots-TiO_2_ nanotube array heterostructures as efficient visible light photocatalysts for photoredox applications J Mater Chem A 2013 1 12229 12238 10.1039/c3ta12856c

[B41] Lo YS Choubey RK Yu WC Hsu WT Lan CW Shallow bath chemical deposition of CdS thin film Thin Solid Films 2011 520 217 223 10.1016/j.tsf.2011.07.035

[B42] Lokhande CD Sankapal BR Pathan HM Muller M Giersig M Tributsch H Some structural studies on successive ionic layer adsorption and reaction (SILAR)-deposited US thin films Appl Surf Sci 2001 181 277 282 10.1016/S0169-4332(01)00392-0

[B43] Tao L Xiong Y Liu H Shen WZ High performance PbS quantum dot sensitized solar cells via electric field assisted in situ chemical deposition on modulated TiO_2_ nanotube arrays Nanoscale 2014 6 931 938 10.1039/c3nr04461k 24281658

[B44] Ke WJ Fang GJ Lei HW Qin PL Tao H Zeng W Wang J Zhao XZ An efficient and transparent copper sulfide nanosheet film counter electrode for bifacial quantum dot-sensitized solar cells J Power Sources 2014 248 809 815

[B45] Singh VP Linam DL Dils DW McClure JC Lush GB Electro-optical characterization and modeling of thin film CdS-CdTe heterojunction solar cells Sol Energ Mat Sol C 2000 63 445 466 10.1016/S0927-0248(00)00063-5

[B46] Raffaelle RP Forsell H Potdevin T Friedfeld R Mantovani JG Bailey SG Hubbard SM Gordon EM Hepp AF Electrodeposited CdS on CIS pn junctions Sol Energ Mat Sol C 1999 57 167 178 10.1016/S0927-0248(98)00172-X

[B47] Kariper A Güneri E Göde F Gümüş C Özpozan T The structural, electrical and optical properties of CdS thin films as a function of pH Mater Chem Phys 2011 129 183 188 10.1016/j.matchemphys.2011.03.070

[B48] Wang H Bai YS Zhang H Zhang ZH Li JH Guo L CdS quantum dots-sensitized TiO_2_ nanorod array on transparent conductive glass photoelectrodes J Phys Chem C 2010 114 16451 16455 10.1021/jp104208z

[B49] Mohanty I Routray CK Singh UP Influence of oxygen during deposition on chemically deposited CdS film Thin Solid Films 2013 527 147 150

[B50] Nishino J Chatani S Uotani Y Nosaka Y Electrodeposition method for controlled formation of CdS films from aqueous solutions J Electroanal Chem 1999 473 217 222 10.1016/S0022-0728(99)00250-8

[B51] Xing JH Xia ZB Hu JF Zhang YH Zhong L Growth and crystallization of titanium oxide films at different anodization modes J Electrochem Soc 2013 160 C239 C246 10.1149/2.070306jes

[B52] Horner G Johne P Kunneth R Twardzik G Roth H Clark T Kisch H Heterogeneous photocatalysis, part XIX - semiconductor type a photocatalysis: role of substrate adsorption and the nature of photoreactive surface sites in zinc sulfide catalyzed C-C coupling reactions Chem-Eur J 1999 5 208 217 10.1002/(SICI)1521-3765(19990104)5:1<208::AID-CHEM208>3.0.CO;2-0

[B53] Ardalan P Brennan TP Lee HBR Bakke JR Ding IK McGehee MD Bent SF Effects of self-assembled monolayers on solid-state CdS quantum dot sensitized solar cells ACS Nano 2011 5 1495 1504 10.1021/nn103371v 21299223

[B54] Kuznetsov AS Tikhomirov VK Moshchalkov VV UV-driven efficient. White light generation by Ag nanoclusters dispersed in glass host Mater Lett 2013 92 4 6

[B55] Chang CY Tsai FY Efficient and air-stable plastics-based polymer solar cells enabled by atomic layer deposition J Mater Chem 2011 21 5710 5715 10.1039/c0jm04066e

[B56] Sapra S Mayilo S Klar TA Rogach AL Feldmann J Bright white-light emission from semiconductor nanocrystals: by chance and by design Adv Mater 2007 19 569 10.1002/adma.200602267

[B57] Shang CQ Dong SM Wang S Xiao DD Han PX Wang XG Gu L Cui GL Coaxial NixCo_2_x(OH)_6x_/TiN nanotube arrays as supercapacitor electrodes ACS Nano 2013 7 5430 5436 10.1021/nn401402a 23647174

[B58] Shan Y Xu JJ Chen HY Electrochemiluminescence quenching by CdTe quantum dots through energy scavenging for ultrasensitive detection of antigen Chem Commun 2010 46 5079 5081 10.1039/c0cc00837k 20559593

