# Supplementary material for: A composite CdS thin film/TiO2 nanotube structure by ultrafast successive electrochemical deposition toward photovoltaic application
Source: Nanoscale Res Lett. 2014 Nov 25;9(1):631. doi: 10.1186/1556-276X-9-631 (PMC4266500; doi:10.1186/1556-276X-9-631)
Supplement: Additional file 1 — Supporting information. This file contains the schematic diagram of the device, the waveform of the applied voltage, the pictures of outcome materials, the relationship of film thickness and growth conditions, and the EDX data on material contents under different conditions, and J-V characteristics of as-fabricated back-side-illuminated solar cells. [file 1556-276X-9-631-S1.pdf]

Supporting Information for "A Composite CdS Thin Film/TiO_2 Nanotube Structure by Ultrafast Successive Electrochemical Deposition towards Photovoltaic Application"

Han Fu, [a] Hong Liu,[*a] Wenzhong Shen[*a]

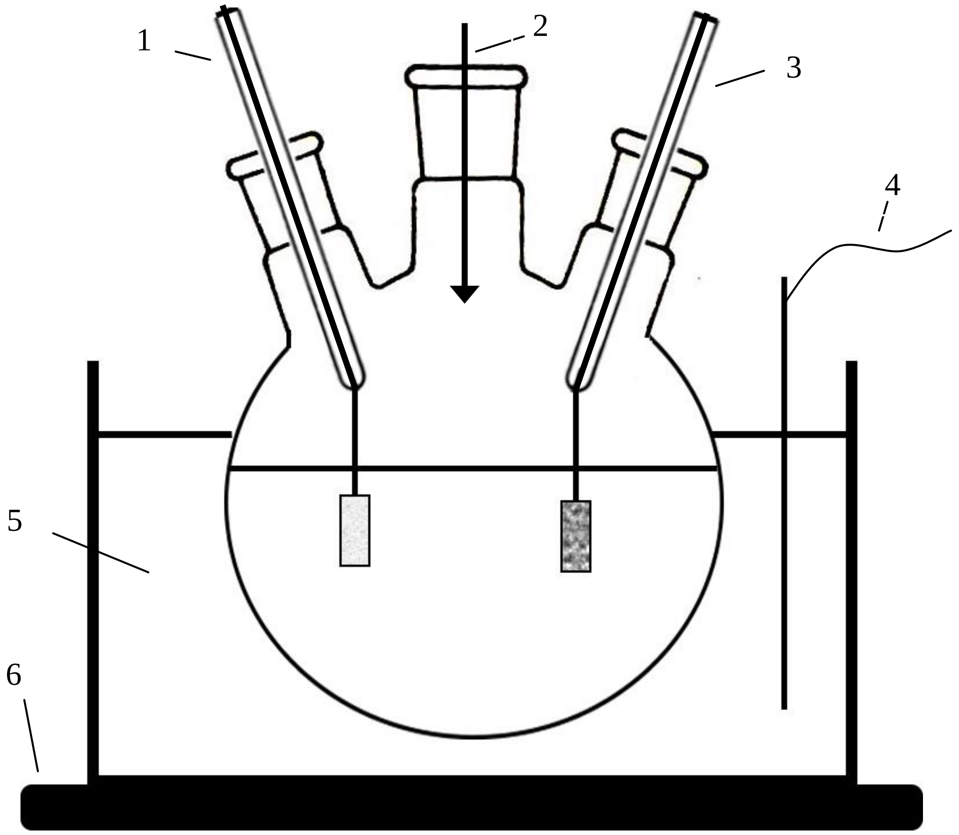

Fig. S1 schematic illustration of experimental setup: (1) Counter electrode; (2) Injection of precursor, (3) Working electrode (4) Thermometer (5) Water bath (6) Heating plate.

The in-situ growth of CdS on substrates was based the equipment as shown in Fig. S1, and the power supply was given by electrochemical workstation.

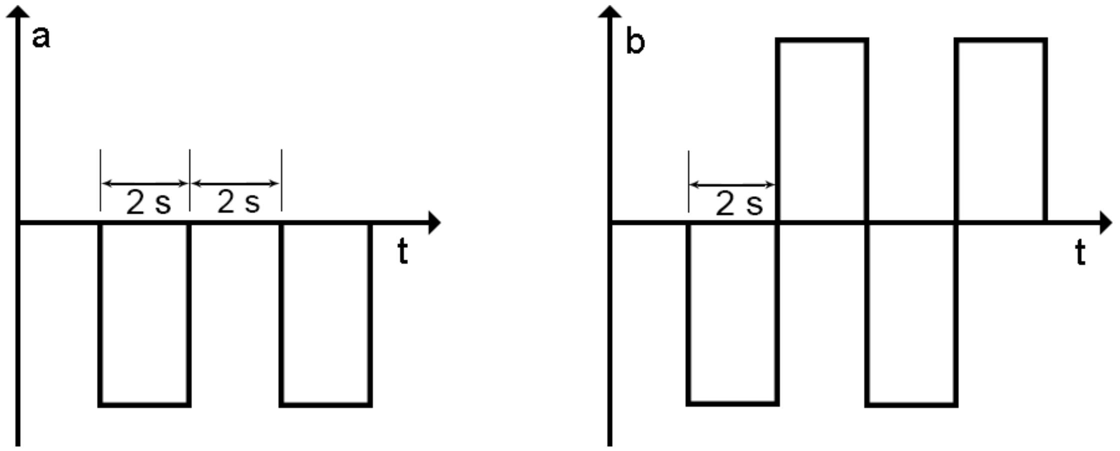

Fig. S2 Schematic illustration of voltage modes: (a) Impulse; (b) AC

The schematic diagram of Impulse voltage, and AC were shown below in Fig. S2. The impulse voltage and AC voltage both have period of 4 s.

`

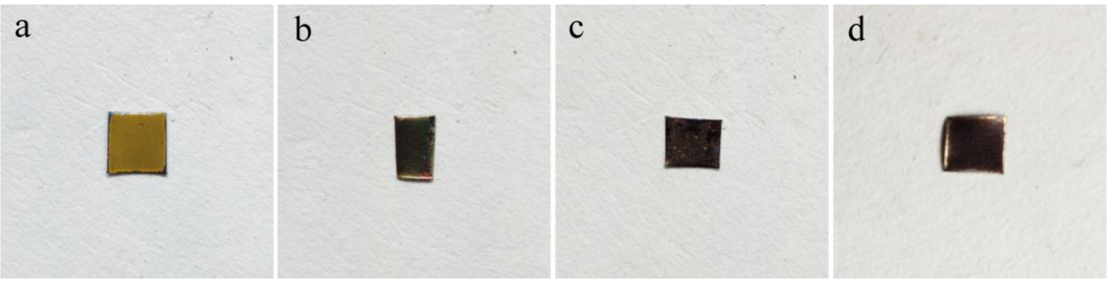

Fig. S3 the picture of CdS coated TNTAs versus different voltage at concentration of Na_2S of 0.001M: (a) |U|=2.5V, (b) |U|=6V, (c) |U|=7V, (d) |U|=10V

We noted that the color of the outcome material will change along with the bias voltage, thus with investigation of XRD, we presented the change of crystal form in Fig. 4. Here are the photographs of three different colors of outcome materials. Figs. S1 (a)-(c) is the CdS coated TNTAs versus alternative voltage 2.5V, 6V, 7V and 10V, and the color the material turns to be lemon yellow, jacinth and light black which correspond to XRD patterns of Fig. 4 a′, c′, d′, f′.

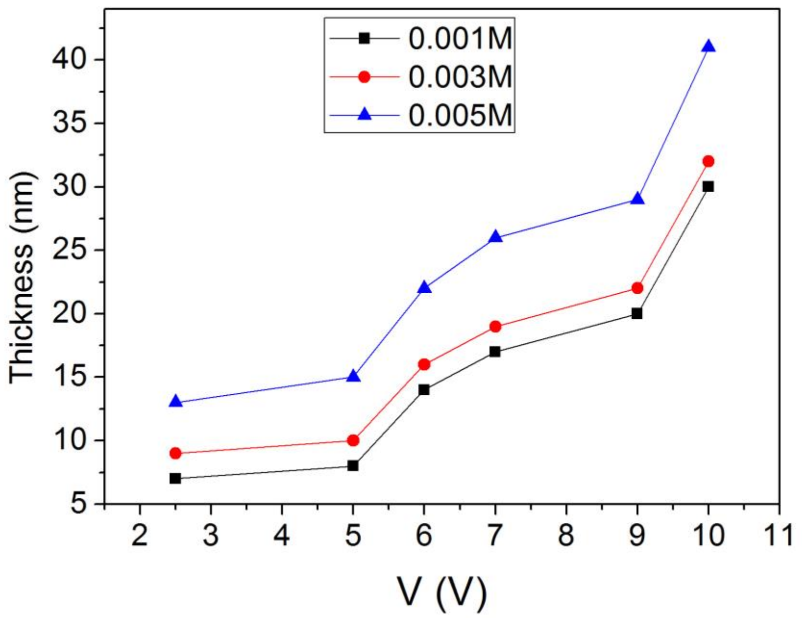

Fig. S4 Relationship of deposited CdS film thickness and the applied voltage magnitude under different precursor concentration (the molar ratio of $Cd(NO_3)_2$: Na_2S is 1:1), in standard growth time (90s).

Generally speaking from the Figure S4, the change of CdS film thickness had three major linear regions: below 5.0V, 5.0-9.0V and 9.0-10.0V. The increase of the thickness with voltage magnitude was small, medium and high, respectively. Considering above 9.0V, the film became too thick that it almost begin to occupy the whole tube, the best controllable region of growth would be the first two regions.

Table S1 EDX index of the CdS coated TiO_2 NP films and NTAs under reaction condition group 1.

Atomic Content (At %)	Element	O K	S K	Ti K	Cd K
	TNP film	77.32	0.45	20.40	1.83
	TNTAs	32.42	10.98	43.41	13.19

Table S2 EDX data showing existence of oxide under 9.0 and 10.0V (with precursor concentration 0.001M).

Voltage (V)	Cd (at%)	S (at%)	Ti (at%)	O (at%)	O:Ti
**2.5**	1.900	1.835	32.000	64.265	2.008
**5.0**	2.230	2.153	31.840	63.777	2.003
**6.0**	4.200	4.000	30.530	51.270	1.679
**7.0**	5.600	5.320	29.600	59.480	2.009
**9.0**	6.000	5.600	29.330	59.070	2.013
**10.0**	8.000	7.480	28.000	56.520	2.018

As shown by the table, the atomic ratio of O:Ti at different voltage was very close to 2, which corresponds to the O content in anodized TiO_2 substrate. The 1.679 value for 6.0V should be due to O vacancies, which is normal for anodic TiO_2 nanotubes. Except that, the average ratio was 2.007. However at 9.0V and 10.0V, the atomic ratio of O to Ti was 2.13 and 2.18, respectively, which indicates slight increase of O content than previous average value in the as-formed material under those conditions. If comparing with the XRD spectra, this slight increase of O content was very likely due to formation of CdO content. Finally, the content of the CdO can be estimated by the ratio of excessive O compared to Cd, which are: at 9.0V, (59.070-29.330*2.007)/6.000=3.4%; at 10.0V, (56.520-28.000*2.007)/8.000=4.1%.

Table S3 Cd and S content and relative ratio under different voltage magnitude and Na_2S concentration.

Voltage (V)	$[Na_2S]$ 0.001M			$[Na_2S]$ 0.003M			$[Na_2S]$ 0.005M		
	Cd (at%)	S (at%)	Cd/S	Cd (at%)	S (at%)	Cd/S	Cd (at%)	S (at%)	Cd/S
2.5	1.900	1.835	1.035	3.600	3.476	1.036	5.100	4.925	1.036
5.0	2.230	2.153	1.036	4.400	4.249	1.036	5.862	5.660	1.036
6.0	4.200	4.000	1.050	6.380	6.112	1.044	7.900	7.568	1.044
7.0	5.600	5.320	1.053	7.216	6.855	1.053	9.173	8.714	1.053
9.0	6.000	5.600	1.071	7.700	7.238	1.064	9.978	9.378	1.064
10.0	8.000	7.480	1.070	9.889	9.246	1.070	12.30	11.50	1.070

Table S4 Photovoltaic characteristic information of the CdS/TiO_2 at different thickness

Thickness (nm)	η (%)	V_{OC} (V)	J_{SC} (mA)	FF
**QDSSC**	0.4641	0.44331	3.1453	0.33285
**18nm**	0.6271	0.52777	3.4600	0.34336
**19nm**	0.7843	0.53266	4.2595	0.34562
**25nm**	1.4300	0.54915	7.3936	0.35221
**28nm**	1.0931	0.53272	5.8436	0.35115
